# Hepatic fat content is a determinant of metabolic phenotypes and increased carotid intima-media thickness in obese adults

**DOI:** 10.1038/srep21894

**Published:** 2016-02-23

**Authors:** Huijie Zhang, Zhimin Ma, Lingling Pan, Yanfang Xu, Jin Shao, Zhufeng Huang, Zheng Chen, Qian Sun, Changqin Liu, Mingzhu Lin, Shuyu Yang, Xuejun Li

**Affiliations:** 1 Department of Endocrinology and Diabetes, The First Affiliated Hospital, Xiamen University, Xiamen, China; 2 Department of Epidemiology, Tulane University Health Sciences Center, New Orleans, LA; 3 Department of Endocrinology, the Second Affiliated Hospital of Soochow University, Suzhou, China; 4 Shanghai Institute of Endocrinology and Metabolism, Department of Endocrine and Metabolic Diseases, Shanghai Clinical Center for Endocrine and Metabolic Diseases, Ruijin Hospital, Shanghai Jiao Tong University School of Medicine, Shanghai, China; 5 Department of Nephrology, The First Affiliated Hospital, Fujian Medical University, Fuzhou, China

## Abstract

Individuals with metabolically healthy obesity (MHO) are at relatively low risk for the development of metabolic abnormalities and subclinical atherosclerosis. This study aims to examine whether hepatic fat accumulation determines metabolic phenotype of obesity and associated with subclinical atherosclerosis. A total of 485 obese adults (aged 40–65 years) who received magnetic resonance spectroscopy were divided into metabolically abnormally obesity (MAO) and MHO groups according to metabolic status. MHO individuals had lower levels of intrahepatic triglyceride (IHTG) content and carotid intima-media thickness (CIMT) than MAO individuals. In multivariable linear regression analyses, IHTG content was independently associated with metabolic syndrome components and CIMT. Based on receiver operating characteristic curve analysis, the IHTG content displayed a higher area under the curve (AUC) for detecting the MAO phenotype (AUC = 0.70, 95%CI = 0.65–0.75) and increased CIMT (AUC = 0.60, 95%CI = 0.54–0.66) than BMI, waist circumference, and body fat percent. MHO individuals were 1.9 times (p < 0.001) more likely to have metabolic syndrome per 1 SD change in IHTG content in multivariable-adjusted models. Likewise, the risk for high CIMT increased 29% per 1 SD change in IHTG content [OR (95% CI):1.29(1.01–1.64)]. These findings suggest that hepatic fat is a potential predictor of metabolically unhealthy obesity phenotype and subclinical atherosclerosis.

Obesity is accompanied by a high incidence of type 2 diabetes mellitus and cardiovascular disease (CVD)^1^,^2^. The impact of obesity on the development of CVD is mediated through a number of metabolic abnormalities, such as dyslipidemia, hyperglycemia, and hypertension^2^; however, the incidence of obesity-related metabolic abnormalities varies widely among obese individuals^3^. Recent interest has focused on a subgroup of obese individuals with normal metabolic phenotypes, referred to as metabolically healthy obesity (MHO)^3^,^4^. Increasing evidence suggests that MHO individuals are relatively protected from cardiometabolic disturbances than those with metabolically abnormal obesity (MAO)^5^,^6^.

Since the differentiation between the diverse obese phenotypes may have important implications for targeted preventive strategies in practice, an adequate definition and comprehensive characteristics of MHO subtype are of paramount importance for the stratification of obese individuals. Several characteristics have been reported to explain the apparently less deleterious metabolic profile of MHO subjects^7^. Among them, lower liver enzyme concentrations, lower uric acid, lower inflammatory profile, or higher lipolytic activity have been put forward as determinants of metabolic phenotype^6^,^8^,^9^,^10^.

In addition, it is well recognized that body fat distribution represents an additional independent determinant of obesity-related cardiometabolic disturbances^11^,^12^,^13^. Evidence suggests that individuals with a selective excess of hepatic fat accumulation are at substantially higher risk of being insulin resistant and having a worse cardiovascular risk profile^12^,^14^. Carotid artery intima media thickness (CIMT) is a noninvasive surrogate marker of subclinical atherosclerosis^15^,^16^, and is linked to various traditional risk factors and adverse cardiovascular outcomes^17^,^18^. However, information regarding whether hepatic fat accumulation determines the metabolic phenotype of obesity and is associated with increased CIMT is not available. In the present study, we set out to identify whether hepatic fat content is a determinant of the metabolic phenotype of obesity and its related subclinical atherosclerosis.

## Results

Table 1 summarizes the mean levels of study variables by subtypes of obese subjects. Within the sample, 41.2%(200/485) of participants were metabolically healthy obese. Compared with MAO subjects, MHO subjects had a favorable metabolic profile, including lower levels of BMI, fasting plasma glucose, postprandial glucose, systolic blood pressure, diastolic blood pressure, triglyceride, total cholesterol, LDL-c, and HOMA-IR, and higher levels of HDL-c. Also, MHO subjects had lower CIMT compared with MAO subjects (0.70 ± 0.14 mm vs. 0.76 ± 0.16 mm, p < 0.001). There was no difference in body fat percent between the two groups. Of interest, MHO subjects had lower intrahepatic triglyceride (IHTG) content than MAO subjects (10.5 ± 9.3% vs.16.3 ± 9.9%, p < 0.001). Additionally, MHO subjects had lower levels of uric acid and liver enzymes, including ALT, AST, and GGT, than MAO subject (All p < 0.01).

**Figure 1:**
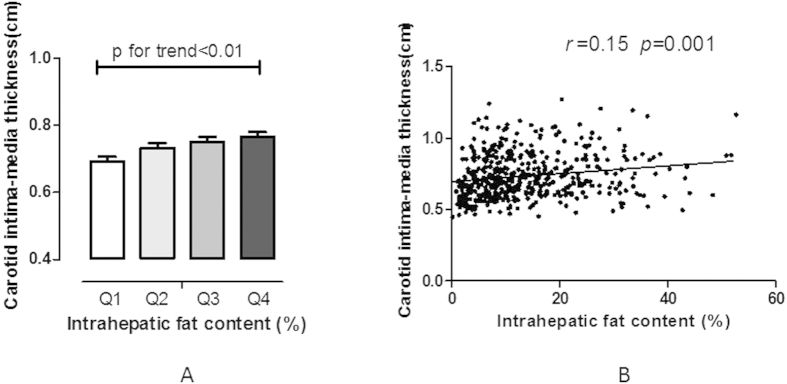
Relationship between intrahepatic triglyceride content (IHTG) and carotid intima-media thickness (CIMT). (**A**) Levels of CIMT by quartile of IHTG; (**B**) correlations of CIMT and IHTG, adjusted for age, gender, and smoking. CIMT = carotid intima-media thickness; IHTG content = intrahepatic triglyceride content.

As shown in Fig. 1, Pearson correlation analysis was performed to investigate the association of IHTG content and CIMT. Results showed that CIMT was significantly positively correlated with IHTG content. By dividing the distribution of IHTG content into quartiles, CIMT gradually increased with the increase in IHTG content (p < 0.001 for trend).

Table 2 presents results of linear regression analyses of metabolic risk factors and IHTG content on MetS and CIMT. In simple linear regression models, IHTG content and HOMA-IR were significantly associated with MetS components. IHTG content was also significantly associated with CIMT, while BMI and HOMA-IR showed no significant association with CIMT. In multivariable linear regression models, BMI, HOMA-IR and IHTG content were all significantly associated with MetS components, after adjustment for age, gender, current smoking, alcohol consumption, physical activity, BMI, hypertension, fasting plasma glucose, triglyceride, HDL-c, and HOMA-IR. Meanwhile, only IHTG content was significantly associated with CIMT.

Table 3 presents results of area under the curve (AUC) calculations for detecting metabolic abnormities and increased CIMT. Based on ROC curve analysis, IHTG content displayed a significantly higher AUC for detecting the MAO phenotype (AUC = 0.70, 95%CI = 0.65–0.75) than BMI, waist circumference, and total body fat. Additionally, IHTG content also displayed a significantly higher AUC for detecting increased CIMT (AUC = 0.60, 95%CI = 0.54–0.66) than total body fat.

The multivariable-adjusted odds ratios (ORs) for the association between IHTG content and MetS or increased CIMT are shown in Table 4. MHO subjects were 1.86 times(p < 0.001) more likely to have MetS per 1 SD increase in IHTG content, after adjustment for age, gender, current smoking, alcohol consumption, physical activity, and BMI, and this relationship remained significant after further adjusting for HOMA-IR and total body fat [OR(95% CI):1.46(1.13–1.88)]. Likewise, IHTG content was significantly associated with increased CIMT, after adjusting for age, gender, current smoking, alcohol consumption, physical activity, BMI, HOMA-IR, and total body fat. After further adjusting for metabolic components, the risk for high CIMT significantly increased by 29% per 1 SD change in IHTG content [OR (95% CI):1.29(1.01–1.64)].

## Discussion

Numerous studies have reported that MHO subjects were at low risk of obesity-related cardiometabolic disturbances^5^,^6^. However, comprehensive characteristics of the MHO subtype are not well determined. In particular, information regarding whether hepatic fat accumulation determines the metabolic profiles of distinct subtypes of obesity, in relation to subclinical CVD profile, is not available. In the present study, the MHO subtype had significantly lower levels of CIMT and IHTG content than the MAO subtype. Furthermore, IHTG content was independently associated with metabolic abnormalities and increased CIMT in obese adults. This study provides strong evidence that hepatic fat accumulation plays a role as a considerable discriminator between MAO and MHO subtypes in relation to increased CIMT.

Evidence has suggested that MHO individuals were relatively protected from CVD as compared to MAO individuals^5^,^6^. In a 15-year follow-up study, subjects with the MHO phenotype did not have increased CVD mortality and all-cause mortality^19^. Ki-Chul Sung *et al.* reported that, among 14,384 South Koreans, MHO individuals had no increased risk of pre-clinical atherosclerosis determined by CAC score^20^. Marini and colleagues suggested that MHO subjects had an intermediate cardiovascular risk profile that fell between MAO subjects and healthy non-obese subjects^5^. In contrast, several studies have questioned the apparently healthy metabolic profile of MHO, showing that it may not translate into lower morbidity and mortality^4^,^21^,^22^. Our data support a lower risk of atherosclerosis among MHO individuals than MAO individuals. Accordingly, strategies and treatments for the primary prevention of CVD in the MHO subtype could be relaxed due to lower risk of CVD.

Because the differentiation of the diverse obese phenotypes may have important therapeutic implications, an adequate definition for the stratification of obese phenotypes is of importance. A challenge in evaluating the health implications of the MHO phenotype is the lack of an adequate determination of what factors characterize this phenotype. Several studies have suggested that body fat distribution might be the most prominent factor explaining the variance of metabolic profile of obesity^23^,^24^. A cross-sectional study of 150 postmenopausal women reported that body composition represented a determinant of metabolic subtypes of obesity^24^. In the current study, we found that the MHO subtype had lower hepatic fat content than the MAO subtype. Consistently, Stefan and colleague reported that MHO individuals had lower level of ectopic fat in the liver compared with MHO individuals in 314 subjects^3^. Importantly, our data demonstrated that IHTG content was independently associated with MetS components in obese subjects. Furthermore, our results indicated that IHTG content, but not total body fat, was an independent determinant of metabolic phenotype of obesity.

In addition, elevated fat accumulation in the liver has been shown to be accompanied by atherosclerosis^14^. Consistently, we found that IHTG content was independently associated with CIMT, irrespective of total body fat. Of note, individuals were 1.29 times more likely to have increased CIMT per 1 SD increase in IHTG content. Indeed, excess hepatic fat accumulation results from the inability of adipose tissue to appropriately store excess energy^11^; thereby it is no surprise that hepatic fat content could predict metabolic profile of obesity. On the other hand, excess hepatic fat accumulation leads to increased expression of multiple inflammatory factors and adipocytokines, which are associated with increased risk of CVD^11^. Therefore, our data suggested that increased hepatic fat accumulation may determine metabolic phenotype with subclinical atherosclerosis in obese adults.

Increased liver enzymes and uric acid concentrations have also been proposed as metabolic factors explaining the differences between MHO and MAO subtype^10^,^24^,^25^. Consistently, we found that MHO subjects had lower levels of serum ALT, AST, GGT, and uric acid than MAO subjects. On the other hand, previous studies indicated that increased hepatic fat accumulation was associated with elevated liver enzymes (i.e. ALT, AST or GGT) and serum uric acid^24^,^26^,^27^. Thus, our data supported the notion that hepatic fat accumulation represented a paramount determinant of metabolic phenotypes of obesity.

This community-based, cross-sectional study provided an opportunity to determine the role of hepatic triglyceride content in predicting the MAO phenotype and subclinical atherosclerosis. There are several limitations to the current study. First, given its cross-sectional design, it is not possible to determine a causal relationship among hepatic fat accumulation and the development of the MAO phenotype and increased CIMT. Second, hepatic triglyceride content was determined by ^1^H-MRS measurement, instead of biopsy-proven steatosis, steatohepatitis, or fibrosis. However, hepatic triglyceride content is a more sensitive measure of fat accumulation in the liver^28^.

In conclusion, out data suggested that hepatic fat content represented an independent determinant of the MHO phenotype. In particular, hepatic fat content was independently associated with MetS and increased CIMT. These results support the notion that hepatic fat accumulation predicts the MAO phenotype and subclinical atherosclerosis. These findings underscore the role of hepatic fat accumulation in the identification of high risk obese phenotypes and the importance of controlling fatty liver for the prevention of CVD.

## Methods

### Study participants

We recruited participants from the Lianqian community, Xiamen, China from April 2011 to December 2013. The details of the study design and methods have been previously reported^29^. In brief, a total of 485 adult obese subjects (waist circumference ≥ 90 cm for men or 80 cm for women) who were randomly chosen to receive magnetic resonance spectroscopy (^1^H-MRS) for the measurement of hepatic fat content were included in the analysis. All participants completed a uniform questionnaire including histories of diabetes, malignancy, cardiovascular disease, smoking status, alcohol consumption, and physical activity. The following participants were excluded: 1) those with any clinical evidence of cirrhosis, biliary obstructive diseases, or other secondary chronic liver diseases (e.g. alcohol intake ≥140 g/week for men or 70 g/week for women currently or in the past 6 months, acute or chronic viral hepatitis, autoimmune hepatitis and/or the use of hepatotoxic medications, such as corticosteroids) and 2) those with established or newly diagnosed type 2 diabetes. The subjects were stratified into metabolically healthy obesity (MHO) and metabolically abnormal obesity (MAO) groups. MHO subjects were defined as those with obesity and one or no metabolic abnormality; MAO subjects were defined as those who were obese and had at least 2 of the following metabolic abnormalities: glucose concentrations ≥5.6 mmol/L or antidiabetes medication use; systolic blood pressure ≥130 mmHg, diastolic blood pressure ≥5 mmHg, or antihypertensive medication use; triglyceride concentrations ≥1.7 mmol/L; and HDL-cholesterol levels <1.03 mmol/L for men and <1.29 mmol/L for women or lipid-lowering medication use as reported previously^30^,^31^.

Written informed consent was obtained from each participant. The study protocol was approved by the Institutional Review Board of the First Affiliated Hospital of Xiamen University. The methods were carried out in accordance with the approved guidelines.

### Clinical and biochemical measurements

Anthropometric measurements included height, weight, waist circumference, and blood pressure. Body mass index (BMI) was calculated as the weight in kilograms divided by the square of the height in meters. Waist circumference was measured at the level of the umbilicus. Blood pressure (BP) was assessed in triplicate using an electronic sphygmomanometer (OMRON Company).

All blood samples were obtained after 12 h of fasting. Plasma glucose, triglyceride (TG), total cholesterol (TC), low-density lipoprotein cholesterol (LDL-c), high-density lipoprotein cholesterol (HDL-c), and uric acid levels were measured by enzymatic colorimetric methods with a Hitachi 7450 analyzer (Hitachi, Tokyo, Japan).Fasting plasma glucose concentrations and 2-h glucose concentrations were measured using the glucose oxidase method. Low-density lipoprotein cholesterol (LDL-C) was calculated by Friedewald’s formula. Serum ALT and AST were measured by standard enzymatic methods. Serum GGT was measured by the Szasz-Persijn method. Serum insulin concentrations were measured using electrochemiluminiscence immunoassay (Roche Elecsys Insulin Test, Roche Diagnostics, Mannheim, Germany). The homeostasis model assessment of insulin resistance (HOMA-IR) was calculated by the following formula: fasting serum insulin (mIU/L) × fasting plasma glucose (mmol/L)/22.5.

Body fat mass was determined using the HOLOGIC whole body DXA system (Hologic Inc., Bedford, MA). Carotid intima-media thickness (CIMT) was measured with high-resolution ultrasonography as previously described^32^.

### Measurement of hepatic triglyceride content

Intrahepatic triglyceride (IHTG) content was determined by magnetic resonance spectroscopy (^1^H-MRS; Avanto 3.0-T, Siemens AG, Erlangen, Germany). This non-invasive measurement of hepatic triglyceride content is reproducible. Images of a sagittal, coronal, and axial cube of 2 cm^3^ volume in the right lobe of the liver were acquired. Quantification of the spectra (water and methylene resonances) was performed as described previously^33^,^34^. Areas of resonance from water protons and methylene groups in fatty acid chains were obtained with a time-domain nonlinear fitting routine using commercial software (Syngo spectroscopy VB15, Siemens AG). The percent of IHTG content was calculated as the ratio of the area under the resonance peak for methylene groups in fatty acid chains of IHTG and the combined area under the resonance peaks for methylene groups and water.

### Statistical analysis

All statistical analyses were performed with SAS version 9.3 (SAS Institute, Cary, NC). Metabolic syndrome (MetS) was defined according to International Diabetes Federation (IDF) diagnostic criteria^35^. These criteria included: 1)central obesity defined as waist circumference ≥90 cm in men or ≥80 cm in women; 2)plus two or more of the following: (a) low serum HDL-c (HDL-c < 1.03 mmol/L in men or <1.29 mmol/L in women), (b) hypertriglyceridemia (TG ≥ 1.7 mmol/L), (c) hypertension (BP ≥ 130/85 mmHg or treatment of previously diagnosed hypertension), or (d) dysglycemia (fasting plasma glucose ≥5.6 mmol/L or previously diagnosed type 2 diabetes).Increased CIMT was defined as average CIMT ≥ 0.8 mm^36^,^37^.The subjects were classified into four quartiles according to IHTG content(Quartile 1, <6.5%, Quartile 2, 6.5–11.0%, Quartile 3, 11.0–19.5%, and Quartile 4, ≥19.5%).

The χ^2^-test was used for comparison of categorical variables between groups. Analyses of covariance were performed using general linear models (GLM) to test differences in study variables between MHO and MAO groups or different quartiles of IHTG contents. The correlation of IHTG content with CIMT was analyzed by Pearson correlation. Receiver operating characteristic (ROC) curve analysis was applied to evaluate the utility of several anthropometric measures, body fat, and IHTG content to determine metabolic phenotypes and subclinical atherosclerosis. Multivariable linear regression models were used to examine the association between IHTG content and CIMT or individual MetS components as continuous variables, adjusted for age, sex, smoking, alcohol consumption, physical activity, BMI, hypertension, glucose, triglyceride, HDL-c, and HOMA-IR. Multivariable logistic regression models were used to examine the association between IHTG content and MetS or increased CIMT, adjusted for age, gender, smoking, alcohol consumption, physical activity, BMI, HOMA-IR, body fat and metabolic components.

## Additional Information

**How to cite this article**: Zhang, H. *et al.* Hepatic fat content is a determinant of metabolic phenotypes and increased carotid intima-media thickness in obese adults. *Sci. Rep.* **6**, 21894; doi: 10.1038/srep21894 (2016).

## Figures and Tables

**Table 1:** Clinical and blood biochemical characteristics by subtypes of obese subjects.

Variables	Overall	Metabolically healthy obesity	Metabolically abnormal obesity	P-value
Sample size	485	200	285	
Age (years)	54.1 ± 7.1	53.4 ± 7.2	54.5 ± 7.1	0.082
Gender (female, n, %)	352 (73)	152 (76)	200 (70)	0.157
BMI (kg/m^2^)	27.1 ± 2.7	26.4 ± 2.6	27.5 ± 2.7	<0.001
Waist circumference (cm)	94.1 ± 6.7	92.9 ± 6.3	94.9 ± 6.8	0.001
Current smokers (n, %)	74(15)	30(15)	44(15)	0.895
Current alcohol drinking (n, %)	133(27.4)	58(29.0)	75(26.3)	0.514
Physical activity (Met/h. week)	8.0(23.1–46.2)	23.6(8.8–52.3)	23.1(7.7–46.2)	0.768
Systolic BP (mmHg)	129.5 ± 16.1	121.4 ± 13.5	135.2 ± 15.4	<0.001
Diastolic BP (mmHg)	77.7 ± 10.1	73.2 ± 8.5	80.9 ± 9.9	<0.001
Triglyceride (mmol/L)	1.60(1.17–2.18)	1.19(0.93–1.47)	1.97(1.60–2.65)	<0.001
Total cholesterol (mmol/L)	5.76 ± 1.00	5.56 ± 0.89	5.89 ± 1.04	<0.001
LDL- cholesterol (mmol/L)	3.76 ± 0.98	3.65 ± 0.89	3.84 ± 1.04	0.039
HDL-cholesterol (mmol/L)	1.31 ± 0.26	1.41 ± 0.25	1.23 ± 0.23	<0.001
Fasting glucose (mmol/L)	5.52 ± 0.52	5.27 ± 0.40	5.70 ± 0.51	<0.001
2-h glucose (mmol/L)	7.85 ± 1.93	7.13 ± 1.57	8.36 ± 2.00	<0.001
HOMA-IR	2.84(1.94–4.01)	2.09(1.53–2.95)	3.32(2.46–4.65)	<0.001
ALT (U/L)	25.6 ± 13.6	22.4 ± 12.4	27.9 ± 14.0	<0.001
AST (U/L)	23.6 ± 6.8	22.8 ± 6.1	24.2 ± 7.2	0.027
GGT (U/L)	35.5 ± 23.1	30.8 ± 20.6	38.8 ± 24.2	<0.001
Uric acid (mmol/L)	346.6 ± 92.0	318.1 ± 83.4	366.6 ± 92.6	<0.001
Body fat percent (%)	33.8 ± 5.6	34.0 ± 5.4	33.7 ± 5.8	0.594
CIMT(mm)	0.73 ± 0.16	0.70 ± 0.14	0.76 ± 0.16	<0.001
IHTG content (%)	13.9 ± 10.1	10.5 ± 9.3	16.3 ± 9.9	<0.001

CIMT = carotid intima-media thickness; BMI = body mass index; HOMA-IR = homeostasis model assessment of insulin resistance; IHTG content = intrahepatic triglyceride content;

Data are presented as the mean ± SD or median (interquartile range).

**Table 2:** Standardized regression coefficients of metabolic risk factors and intrahepatic triglyceride content on metabolic syndrome and CIMT.

Independent variables	Unadjusted			Adjusted^a^		
	β	SE	P	β	SE	P
MetS components						
BMI	0.067	0.051	0.189	0.147	0.051	0.004
HOMA-IR	0.401	0.052	<0.001	0.153	0.050	0.003
IHTG content	0.182	0.047	<0.001	0.102	0.045	0.023
Model R^2^(%)	0.209			0.447		
CIMT						
BMI	0.008	0.003	0.333	0.007	0.008	0.412
HOMA-IR	0.011	0.008	0.169	0.007	0.008	0.405
IHTG content	0.021	0.008	0.009	0.016	0.007	0.030
Model R^2^(%)	0.038			0.248		

CIMT = carotid intima-media thickness; IHTG content = intrahepatic triglyceride content; BMI = body mass index; HOMA-IR = homeostasis model assessment of insulin resistance; β = standardized regression coefficient; SE =  standard error.

^a^adjusted for age, sex, smoking, alcohol consumption, physical activity, BMI, hypertension, glucose, triglyceride, HDL-c and HOMA-IR.

**Table 3:** ROC curve analysis for detecting metabolic abnormities and increased CIMT.

Test variables	MAO phenotype			Increased CIMT		
	AUC	95%CI	P	AUC	95%CI	P
BMI	0.62	0.57–0.67	<0.001	0.56	0.50–0.62	<0.001
Waist circumference	0.59	0.54–0.64	<0.001	0.56	0.50–0.62	<0.001
Body fat	0.49^d^,^e^	0.44–0.54	<0.001	0.44^d^,^e^	0.38–0.50	<0.001
IHTG content	0.70^a^,^b^,^c^	0.65–0.75	<0.001	0.60^c^	0.54–0.66	<0.001

ROC = receiver operating characteristic; MAO = metabolically abnormal obesity; CIMT = carotid intima-media thickness; IHTG content = intrahepatic triglyceride content; BMI = body mass index; AUC = area under the curve; CI = confidence interval;

^a^p value < 0.05 for the comparison AUC of IHTG content versus AUC of BMI;

^b^p value < 0.05 for the comparison AUC of IHTG content versus AUC of waist circumference;

^c^p value < 0.05 for the comparison AUC of IHTG content versus AUC of body fat;

^d^p value < 0.05 for the comparison AUC of BMI versus AUC of body fat;

^e^p value < 0.05 for the comparison AUC of Waist circumference versus AUC of body fat.

**Table 4:** Odds ratios for increased CIMT and metabolic syndrome according to hepatic triglyceride content.

	Metabolic syndrome		Increased CIMT	
	OR(95% CI)	P-value	OR(95% CI)	P-value
Model 1	1.86(1.46–2.37)	<0.001	1.31(1.05–1.63)	0.017
Model 2	1.46(1.13–1.88)	0.004	1.27(1.01–1.60)	0.043
Model 3	—	—	1.29(1.01–1.64)	0.045

OR = odds ratio; CI = confidence interval; BMI = body mass index; HOMA-IR = homeostasis model assessment of insulin resistance; CIMT = carotid intima-media thickness;

Model 1: adjusted for age, gender, smoking, alcohol consumption, physical activity, and BMI.

Model 2: Model 1+adjusted for HOMA-IR and body fat.

Model 3: Model 2+adjusted for metabolic components, including glucose, triglyceride, HDL-c and blood pressure.
